# The value of ischemia-modified albumin compared with d-dimer in the diagnosis of pulmonary embolism

**DOI:** 10.1186/1465-9921-9-49

**Published:** 2008-05-30

**Authors:** Suleyman Turedi, Abdulkadir Gunduz, Ahmet Mentese, Murat Topbas, Suleyman C Karahan, Selman Yeniocak, Ibrahim Turan, Oguz Eroglu, Utku Ucar, Yunus Karaca, Suha Turkmen, Robert M Russell

**Affiliations:** 1 Department of Emergency Medicine, Karadeniz Technical University Faculty of Medicine, Trabzon, Turkey; 2 Department of Biochemistry, Karadeniz Technical University Faculty of Medicine, Trabzon, Turkey; 3 Department of Public Health, Karadeniz Technical University Faculty of Medicine, Trabzon, Turkey

## Abstract

**Study objective:**

The primary aim of this study was to investigate whether IMA levels are helpful in the diagnosis of pulmonary embolism (PE). The secondary aim was to determine whether IMA was more effective alone or in combination with clinical probability scores in the diagnosis of PE. Thirdly, the sensitivity and specificity of IMA is compared with D-dimer both with and without clinical probability scores in patients with suspected PE.

**Methods:**

Consecutive patients presenting to the emergency department with suspected PE were prospectively recruited, and healthy volunteers were also enrolled as controls. D-dimer and IMA levels were measured for the entire study group. Wells and Geneva scores were calculated and s-CTPA was performed on all suspected PE patients.

**Results:**

The study population consisted of 130 patients with suspected PE and 59 healthy controls. Mean IMA levels were 0.362 ± 0.11 ABSU for Group A, the PE group (n = 75); 0.265 ± 0.07 ABSU for Group B, the non-PE group (n = 55); and 0.175 ± 0.05 ABSU for Group C, the healthy control group (p < 0.0001). At a cut-off point of 0.25 ABSU, IMA was 93% sensitive and 75% specific in the diagnosis of PE. PPV was 79.4% and NPV was 78.6%. Mean D-dimer levels were 12.48 ± 10.88 μg/ml for Group A; 5.36 ± 7.80 μg/ml for Group B and 0.36 ± 0.16 μg/ml for Group C (p < 0.0001). The D-dimer cut-off point was 0.81 μg/ml with a sensitivity of 98.9% and a specificity of 62.7%, PPV of 69.4% and NPV of 83.3%. The use of IMA in combination with Wells and Geneva clinical probability scores was determined to have a positive impact on these scores' sensitivity and negative predictive values.

**Conclusion:**

IMA is a good alternative to D-dimer in PE diagnosis in terms of both cost and efficiency. Used in combination with clinical probability scores, it has a similar positive effect on NPV and sensitivity to that of D-dimer. The PPV of IMA is better than D-dimer, but it is still unable to confirm a diagnosis of PE without additional investigation.

## Introduction

### Background

Pulmonary embolism (PE) is a common and potentially life-threatening disorder. Because symptoms and signs are nonspecific, the diagnosis of PE in the emergency department (ED) still poses difficulties [1].

### Importance

None of the available laboratory tests are capable of reliably excluding the diagnosis, and the clinician therefore has to rely on such diagnostic techniques as lung scintigraphy, spiral computed tomographic pulmonary angiography (s-CTPA) or pulmonary angiography [2]. New and simple tests are therefore needed in order to exclude PE and to reduce the number of these sophisticated imaging techniques required.

Under acute ischemic conditions, the metal binding capacity of albumin to transition metals such as copper, nickel and cobalt is reduced, generating a metabolic variant of the protein generally referred to as ischemia modified albumin (IMA) [3]. IMA is a sensitive marker of myocardial ischemia, skeletal ischemia, mesenteric ischemia and stroke [4-7]. There is only one study in the literature regarding the diagnostic value of IMA in the diagnosis of PE [8].

### Objectives

The primary aim of our study was to investigate whether IMA levels are a useful marker that can be used in the diagnosis of PE, the secondary aim being to determine whether IMA alone or in combination with clinical probability scores can be used as an alternative to D-dimer as a laboratory marker in the diagnostic work-up of patients with suspected PE.

## Materials and methods

### Study design

This study is a primary analysis of data collected during a prospective, observational study at a tertiary care center.

### Setting

The setting was the ED of Karadeniz Technical University, Faculty of Medicine in the city of Trabzon in Turkey. Some 20,000 patients present to the ED annually.

### Selection of participants

The protocol for the study was approved by the hospital's local ethical committee. Patients were included in the study if the emergency physician suspected PE. One hundred forty-seven consecutive patients presenting to the ED with suspected PE from April, 2006, to April, 2007, were prospectively recruited for the study. Additionally, fifty-seven healthy volunteers served as a reference for biochemical parameters.

Exclusion criteria were: (i) other acute ischemic diseases newly diagnosed during the ED visit in question, such as acute coronary syndrome (ACS), acute ischemic cerebrovascular disease, acute peripheral arterial occlusion, or acute mesenteric ischemia; (ii) an abnormal serum albumin level making the determination of IMA levels impossible (normal level 3,5–5,5 mg/dl); (iii) advanced liver, kidney or heart failure; (iv) troponin-T and ECG testing was performed for evidence of asymptomatic coronary ischemia; (v) age <18 years; (vi) allergy to contrast material and (vii) refusal to participate in the study. The exclusion criteria applied during the enrolment of the control-healthy group were the same as those for the patient group. Patients were observed until discharged from the hospital or until death.

### Data collection and processing

Emergency physicians completed a questionnaire consisting of details of the patient's medical history (such as risk factors). Physical examination, chest X-ray and ECG, IMA, D-dimer and arterial blood analysis were performed for all patients, and both the simplified Well's and the Geneva scores were calculated before tomographic examination [9,10].

Spiral-CTPA was performed on all patients who had no exclusion criteria. Patients were divided into two groups according to the s-CTPA results, Group A, the PE group, and Group B, the non-PE group. The healthy control group was classified as Group C. s-CTPA was not performed in Group C although blood analysis for IMA and D-dimer was carried out.

## Methods of measurement

### IMA measurement

Blood samples were taken from the brachial vein at time of presentation. Reduced cobalt to albumin binding capacity (IMA level) was analyzed using the rapid and colorimetric method developed by Bar-Or et al. [11]. This is based on the principle of quantitative scanning of the free cobalts present after cobalt binding has taken place. This means that high absorbance levels as a result of increased amounts of free cobalt in the environment can be determined.

### D-dimer measurement

D-dimer was assayed using the quantitative, immuno-tubidimetric STA-Liatest D-dimer kit assay from Diagnostica Stago, Asnieres, France, which was run on an automatic coagulation analyzer (STA-compact, Diagnostica Stago) in a routine setting.

### CT imaging and images interpretation

All patients underwent s-CTPA within 12 hours of selective pulmonary angiography using a 16 detector spiral CT scanner (Siemens Somatom Sensatio, Germany). CT scans were reviewed by radiologists experienced in analyzing s-CTPA. The radiologists were blinded to the results of the D-dimer and IMA testing. The s-CTPA criteria used to diagnose PE consisted of direct visualization of non-occlusive endoluminal thrombus (central filling defect or partially outlined by contrast agent) or of complete occlusion by thrombus in normal-sized or enlarged vessels.

#### Primary data analysis

Statistical analysis was performed using the SPSS version 13.0 (SPSS, Chicago, IL, USA). For the IMA and D-dimer, the test characteristics, sensitivity, specificity, negative and positive predictive values were calculated according to the s-CT results. The area beneath the receiver operating characteristics (ROC) curves was used to compare the discriminative power of the IMA and D-dimer tests in the diagnosis or ruling out of PE. The compatibility of the data with normal distribution was investigated using the Kolmogorov-Smirnov test. Kruskal-Wallis analysis of variance (Mann-Whitney U-test with post-hoc Bonferroni correction) was used to compare IMA and D-dimer among the groups. Statistical significance was assumed at a level of P < 0.05.

## Results

### Characteristics of study subjects

A total of 147 patients with suspected PE and 59 healthy control subjects were enrolled in the study. Seventeen patients were excluded because of predefined criteria: ACS (n = 7), acute ischemic cerebrovascular disorder (n = 1), paradoxical embolism (n = 1), advanced renal insufficiency (n = 3), advanced congestive heart failure (n = 1), peripheral arterial occlusion (n = 3), sepsis (n = 1).

**Figure 1:**
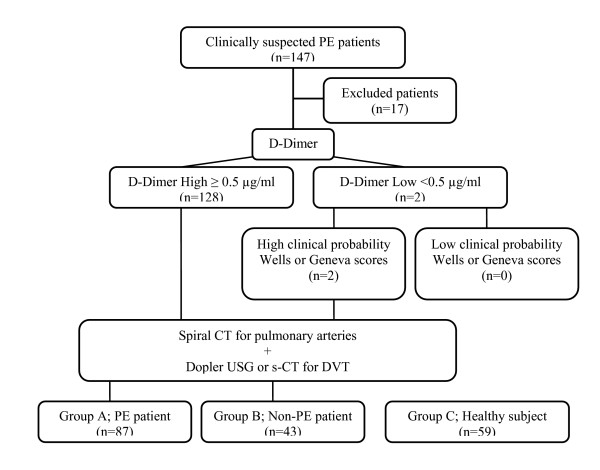
Our diagnostic work-up in suspected PE patients.

All the 130 remaining suspected PE patients underwent s-CTPA at the time of admission. Patients were divided into two groups, Group A, the PE group (n = 75), and Group B, the non-PE group (n = 55), according to the s-CTPA results. The healthy control group was classified as Group C (n = 59). Our diagnostic work-up is presented in Figure 1.

The baseline demographic and clinical characteristics of Groups A, B and C are given in Table 1.

**Table 1:** Baseline demographic and clinical characteristics of the study population.

	Group A (n = 87, 46%)		Group B (n = 43, 22,8%)		Group C (n = 59,31,2%)	
Mean Age ± SD (years)	68.37 ± 11.55		67.30 ± 14.94		62.42 ± 10.46	

Sex	n	%	n	%	n	%
	
Female	53	60.9	20	46.5	29	49.1
Male	34	39.1	23	53.5	30	50.9

Symptoms	n	%	n	%		
			
Cough	27	31.0	14	32.6		
Chest pain	42	48.3	17	39.5		
Dyspnea	77	88.5	35	81.4		
Hemoptysis	8	9.2	4	9.3		
Syncope	18	20.7	5	11.6		
Symptoms of DVT	7	8.0	1	2.3		
Asymptomatic	3	3.4	1	2.3		

Clinical signs	Mean ± SD		Mean ± SD			
			
Heart rate	104.74 ± 23.56		103.88 ± 23.02			
Respiratory rate	27.53 ± 6.26		25.65 ± 7.77			
Fever	36.75 ± 0.79		36.92 ± 0.78			
Systolic BP	113.39 ± 35.57		122.86 ± 28.99			
Diastolic BP	72.44 ± 19.88		75.63 ± 17.85			

Chest X-ray signs	n	%	n	%		
			
Pleural effusion	28	32.2	21	48.8		
Atalectasis	26	29.9	11	25.6		
Diaphragm elevation	26	29,8	11	25.6		
Hampton's sign	4	4.6	0			
Westermach sign	1	1.1	0			
Pulmonary artery dilatation	10	11.5	2	4.7		

ECG signs	n	%	n	%		
			
S1Q3T3	16	18.4	1	2.3		
Sinusal tachycardia	42	48.3	18	41.9		
Atrial fibrillation	14	16.1	8	18.6		
T negativity	9	10.3	3	7.0		
RBBB	2	2.3	0			

Risk factors for VTE	n	%	n	%		
			
Previous DVT	11	12.64	2	4.65		
Previous PE	4	4.59	1	2.32		
Varicose veins	8	9.19	2	4.65		
Chronic venous insuffficiency	7	8.04	1	2.32		
Previous stroke	12	13.79	4	9.30		
COPD	18	20.68	13	30.23		
CHF	27	31.03	20	46.51		
Cancer	9	10.34	2	4.65		
Recent surgery	22	25.28	3	6.97		
Recent trauma	8	9.19	1	2.32		
Immobilization	57	65.51	21	48.83		
Pregnancy	2	2.29	1	2.32		
Post-partum period	0		1	2.32		
Obesity	10	11.49	0			
Long bone fracture	5	5.74	0			

#### Main results

The serum IMA and D-dimer levels of the groups are shown in Table 2.

**Table 2:** The serum IMA and D-dimer levels of the groups.

	Group A	Group B	Group C	P value
IMA levels (ABSU) ± SD	0.362 ± 0.11	0.265 ± 0.07	0.175 ± 0.05	0.0001
D-dimer levels (μg/ml) ± SD	12.48 ± 10.88	5.36 ± 7.80	0.36 ± 0.16	0.0001

**Figure 2:**
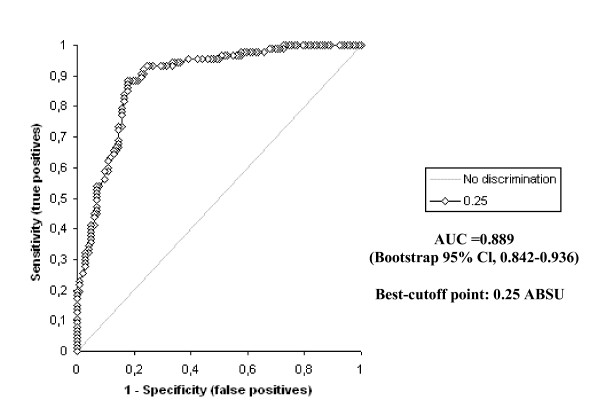
Receiver operating characteristics of ischemia modified albumin levels.

**Figure 3:**
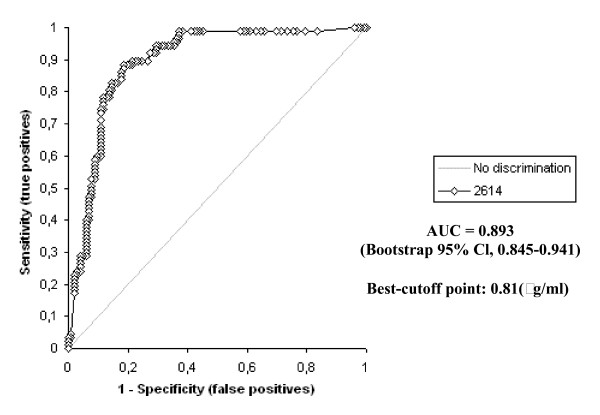
Receiver operating characteristics of D-dimer levels.

The D-dimer vs IMA levels were determined to be statistically different to one another in all groups (p < 0.0001) The highest IMA and D-dimer levels were determined in Group A patients, while there was also a statistically significant difference between the levels in Groups B and C (p < 0.0001). The receiver operating characteristic curve of IMA and D-dimer for diagnosis of PE at admission is shown in Figure 2 and 3.

The area under the curve for IMA was 0.889 (bootstrap 95% Cl, 0,842–0,936). The optimum diagnostic cut-off point maximizing sensitivity and specificity was determined to be 0.25 ABSU, with a sensitivity of 93.1% and a specificity of 75.5%. The corresponding PPV and NPV levels were 79.4% and 78.6%, respectively. The area under the curve for D-dimer was 0.893 (bootstrap 95% Cl, 0,845–0,941). The optimum diagnostic cut-off point maximizing sensitivity and specificity was determined to be 0.81 (μg/ml), with a sensitivity of 98.9% and a specificity of 62.7%. The corresponding PPV and NPV levels were 69.4% and 83.3%, respectively.

**Figure 4:**
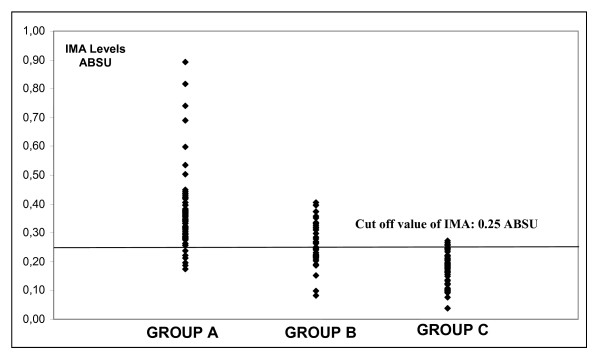
Scattergram of the serum IMA levels in all groups.

**Figure 5:**
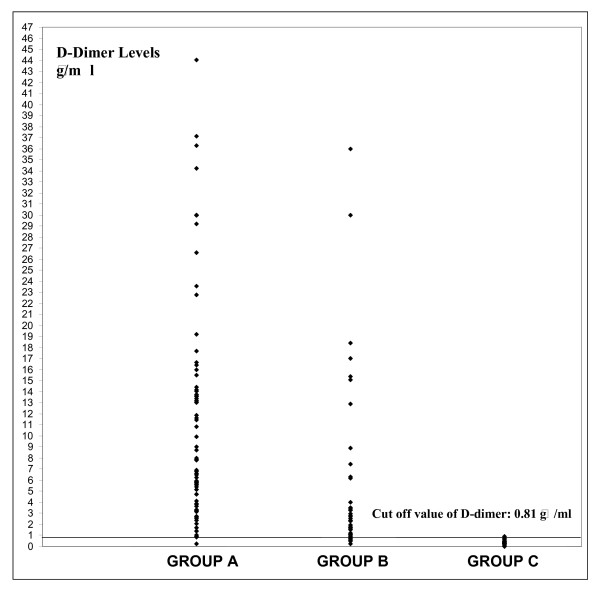
Scattergram of the serum D-dimer levels in all groups.

The scattergram of the serum IMA and D-dimer values in all groups is shown in Figure 4 and 5.

Predictive values of negative IMA (<0.25 ABSU), negative D-dimer (<0.81 μg/ml), CRP ≤ 0.59 mg/dl, Sa02 ≥ 90%, respiratory rate ≤ 20/mi, heart rate ≤ 100/min and PH<7.45, alone and in combination for excluding PE diagnosed on the basis of s-CTPA, are shown in Table 3.

**Table 3:** Predictive value of negative IMA (<0.25 ABSU), negative D-dimer (<0.81 μg/ml), CRP ≤ 0.59 mg/dl, Sa02 ≥ 90%, respiratory rate ≤ 20/min, heart rate ≤ 100/min and PH<7.45, alone and in combination for excluding PE diagnosed on the basis of s-CTPA.

Observation	Correct exclusion	Negative predictive value
Negative IMA (<0.25 ABSU)	22/28	0.786
Negative D-dimer (<0.81 μg/ml)	5/6	0.883
Negative CRP (<0.59 mg/dl)	6/11	0.545
Sa 02 ≥ 90%	30/89	0.337
Respiratory rate ≤ 20/min	8/21	0.381
Heart rate ≤ 100/min,	22/61	0.361
PH<7.45	28/64	0.438
Negative IMA and Sa02 ≥ 90%	15/20	0.750
Negative IMA and respiratory rate ≤ 20/min	5/6	0.833
Negative IMA and heart rate ≤ 100/min,	11/14	0.786
Negative IMA and PH <7.45	22/28	0.786
Negative IMA and negative D-dimer	3/3	1.00
Negative IMA and negative CRP	3/4	0.750
Negative IMA and negative D-dimer and negative CRP	1/1	1.00
Negative IMA and Sa02 ≥ 90% and heart rate ≤ 100/min	0/0	No definition

Group A and B Wells and Geneva clinical probability scores were also analyzed in our study. Group C (healthy volunteers) scores were not included in this analysis, and statistical calculations were performed for Group A and B patients alone.

**Figure 6:**
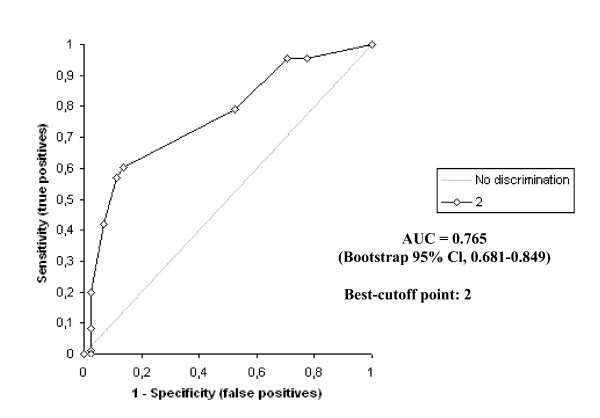
Receiver operating characteristics (ROC) curve of Wells score.

**Figure 7:**
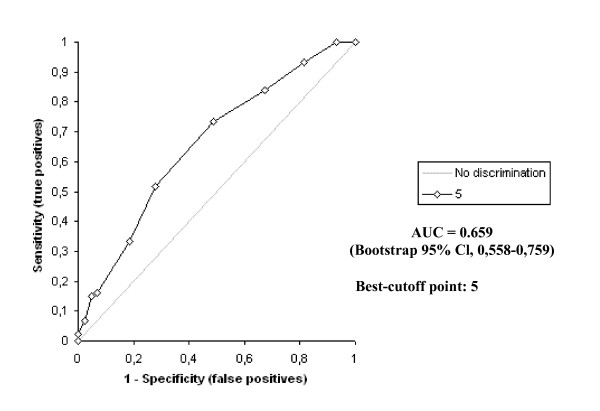
Receiver operating characteristics (ROC) curve of Geneva score.

The receiver operating characteristic curves of Wells and Geneva scores for diagnosis of PE at admission are shown in Figure 6 and 7. According to these,; the area under the curve for the Wells score was 0.765 (bootstrap 95% Cl, 0.681–0.849). The optimum diagnostic cut-off point maximizing sensitivity and specificity was found to be 2 points, with a sensitivity of 79.3% and a specificity of 48.8%. The corresponding PPV and NPV levels were 75.8% and 53.8%, respectively.

The area under the curve for the Geneva score was 0.659 (bootstrap 95% Cl, 0.558–0.759). The optimum diagnostic cut-off point maximizing sensitivity and specificity was found to be 5 points, with a sensitivity of 73.6% and a specificity of 51.2%, respectively. The corresponding PPV and NPV levels were 75.3% and 48.9%, respectively.

Based on the cut-off values determined, biochemical markers and clinical probability scores were subsequently combined. Using a positive s-CTPA as the standard for diagnosis of PE, the diagnostic accuracy levels of the IMA, D-dimer, Well's score, Geneva score, Well's score + IMA, Geneva score + IMA, Well's score + D-dimer, Geneva score + D-dimer are shown in Table 4.

**Table 4:** Performance characteristics of the Wells score, Geneva score, Well's score + IMA and Geneva score + IMA using the cutoff point of pretest probability, based on s-CTPA as reference standard.

Score/IMA	Sensitivity (95% Cl)	Specificity (95% Cl)	PPV (95% Cl)	NPV (95% Cl)
IMA (cutoff 0.25 ABSU)	93.1	52.2	79.4	78.6
D-dimer (cutoff 0.81 μg/ml)	98.9	11.6	69.4	83.3
Wells score (cutoff 2 point)	79.3	48.8	75.8	53.8
Geneva score (cutoff 5 point)	73.6	51.2	75.3	48.9
Wells score and IMA	97.7	23.3	72.0	83.3
Geneva score and IMA	97.7	20.9	71.4	81.8
Wells score and D-dimer	100.0	2.3	67.4	100.0
Geneva score and D-dimer	100.0	4.7	68.0	100.0

## Limitations

Because our facility is a tertiary referral center and receives many patients who have been pre-screened by outlying hospitals and clinics, the prevalence of PE in our patients was very high. The PPV and NPV levels for the tests and criteria described may be different for populations with a different prevalence of PE.

The definitive diagnosis of PE was made using s-CTPA. The sensitivity and specificity of s-CT range from 53% to %100 and from 81% to 100%, respectively, when pulmonary arteriography is used as the standard for diagnosis of PE [12]. For that reason, subsegmental or micro-embolisms invisible under s-CTPA may be present, especially in Group B patients. Even if this is not absolutely certain, it may well account for some high IMA values determined in Group B patients.

There are no data for the period following hospital discharge, so there may have been patients in Group B who developed PE subsequently.

IMA is a new biomarker, the levels of which are significantly influenced by wide range of physiological variables, including exercise and hydration. It may also be elevated in a number of other diseases. In our patient selection, we eliminated patients with advanced liver, kidney or congestive heart failure, which can alter IMA levels. We were unable, however, to check for all variables which could possibly influence IMA levels.

## Discussion

The literature contains studies regarding the use of IMA in acute ischemic conditions. It has been reported in these that IMA can be used as a diagnostic marker, especially in ACS [13-15]. IMA may not be specific for cardiac ischemia. Data concerning IMA levels in non-cardiac ischemia are limited. Some evidence suggests that IMA increases in stroke, mesenteric ischemia, end-stage renal disease, liver disease and some neoplasms [16].

There is only one study in the literature about the use of IMA for diagnostic purposes in PE. This study, by Turedi et al., consisting of 30 patients with PE and 30 healthy individuals, demonstrated that serum IMA levels were significantly higher than those in healthy individuals in 97% of patients [8]. Despite being a limited study, this research by Turedi et al. is an indication that IMA levels might be used in the diagnosis of PE.

Various clinical probability scores and biochemical markers are currently employed in order to exclude suspected PE in patients without using invasive and high-cost scanning tools. The most frequently used among these biochemical markers is D-dimer. The negative predictive value of D-dimer levels for ruling out a diagnosis of thromboembolic disease has been reported as high [17]. However, D-dimer is a rather non-specific marker and its positive predictive value is very low.

The assessment of pretest probability, allowing the categorization of patients clinically suspected of having PE into low, intermediate and high clinical probability, is an essential step in contemporary diagnostic strategies because such categorization helps determine the need for and type of additional testing in patients suspected of having pulmonary emboli [18]. The association of a low or moderate clinical probability with a normal D-dimer assay may confidently rule out PE without using imaging modalities [19]. However, in the presence of a high clinical probability, most clinicians believe that additional tests are necessary [20].

Wells et al. described a first extended score, which was rather complex and not easy to use in daily practice, and subsequently developed a simplified one [9]. The Geneva score has been described by Wicki et al. as a prediction rule [10]. It has been shown in the literature that these clinical probability scores in combination with the use of such biochemical markers as CRP and D-dimer can be useful in excluding PE [21-23]. However, the sensitivity and specificity problems associated with both probability scores and markers such as D-dimer make the identification of new, more accurate markers desirable.

In this study we investigated the diagnostic value of IMA in PE and whether or not IMA levels enhanced the diagnostic accuracy of commonly used risk stratification scales.

With a cut-off value of 0.25 ABSU, IMA sensitivity in the diagnosis of PE was 93.1%, specificity was 75.5%, positive predictive value was 79.4% and negative predictive value was 78.6%. The PPV of a positive IMA is better than the PPV of a positive D-dimer in the diagnosis of PE. D-dimer sensitivity and NPV have been demonstrated to be better than that of IMA and NPV. However, the results for IMA are very close to those for D-dimer and suggest that it can be used as an alternative marker. Used together with clinical probability scores it has a similarly positive effect on NPV and sensitivity to that of D-dimer. In addition IMA is a rapid and low-cost technique. Compared with D-dimer, the costs involved are some 100–200 times lower. In order to reduce patient exposure to radiation and lengthy and high-cost radiological tests, there is a need for economical and high-accuracy markers. Although it cannot be said, on the basis of the results of our study, that IMA is superior to D-dimer for that purpose, it may still be regarded as an alternative to D-dimer in terms of cost and the results determined. More comprehensive studies now need to be carried out on this subject.

In conclusion, IMA is a good alternative to D-dimer in the exclusion of PE diagnosis in terms of cost and efficiency. When used in combination with clinical probability scores, it has a similar positive effect on sensitivity to that of D-dimer and a superior one to that of NPV.

The PPV of IMA is better than D-dimer but is still unable to rule out a diagnosis of PE without additional studies. Wider-ranging studies in the future may mean that IMA becomes an important marker in the diagnostic approach to PE.

## Abbreviations

IMA: ischemia-modified albumin; PE: pulmonary embolism; ED: emergency department; s-CTPA: spiral computed tomographic pulmonary angiography; ABSU: absorbance units; PPV: positive predictive value; NPV: negative predictive value; ACS: acute coronary syndrome; ECG: electrocardiography; DTT: dithiotheitol; SPSS: statistical package for the social sciences; ROC: receiver operating characteristics; DVT: deep vein thrombosis; SD: standard deviation; BP: blood pressure; RBBB: right bundle brunch block; VTE: venous thromboembolism; COPD: chronic obstructive pulmonary disease; CHF: congestive heart failure; AUC: area under the curve; CRP: C-reactive protein; Sa02: oxygen saturation.

## Authors' contributions

ST conceived the study, designed the trial, supervised the conduct of the trial and reviewed the literature. AG conceived the study, designed the trial, supervised the conduct of the trial and reviewed the literature. AM supervised the biochemical data collection and analyzed of these data. MT provided statistical advice on study design and analyzed the data. SCK supervised the conduct of the trial and biochemical data collection. SY in the collection, analysis, and interpretation of data. IT analyzed the biochemical data. OE in the collection, analysis, and interpretation of data. UU analyzed the biochemical data. YK in the collection, analysis, and interpretation of data. ST in the collection, analysis, and interpretation of data. RMR in the writing of the manuscript.
